# Supplementary material for: The Small RNA Universe of Capitella teleta
Source: Front Mol Biosci. 2022 Feb 25;9:802814. doi: 10.3389/fmolb.2022.802814 (PMC8915122; doi:10.3389/fmolb.2022.802814)
Supplement: Supplementary file 1 [file DataSheet1.ZIP › Supplement/known/CAPTEscaffold_84_5893.pdf]

| | | |
|---|---|---|
| Provisional ID | : | CAPTEscaffold_84_5893 |
| Score total | : | 14881.4 |
| Score for star read(s) | : | 3.9 |
| Score for read counts | : | 14871.7 |
| Score for mfe | : | 1.1 |
| Score for randfold | : | 1.6 |
| Score for cons. seed | : | 3 |
| Total read count | : | 29182 |
| Mature read count | : | 29031 |
| Loop read count | : | 0 |
| Star read count | : | 151 |

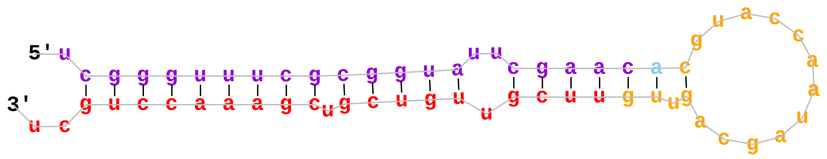


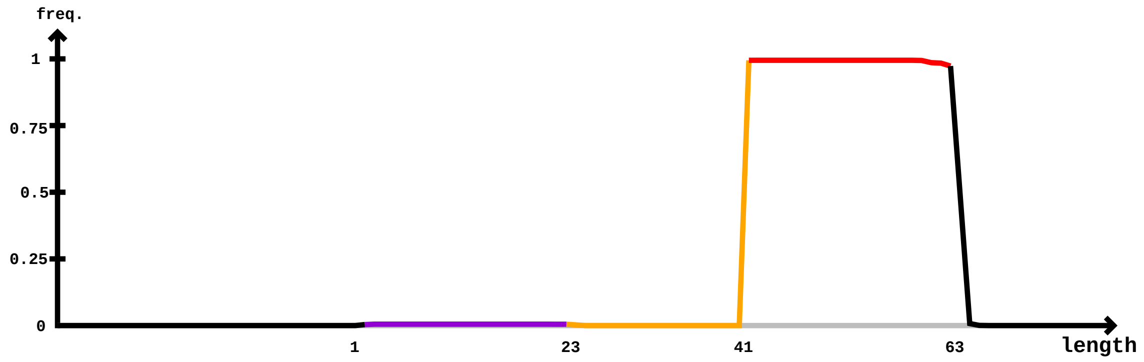


Star                                    Mature

```
5'- auauacuauaccugaccauggucuuucaauucggguuucgcgguauucgaacacguaccaauagcaguuguucguugucgucgaaaccugcuuugaaagauaguuuug  -3'   obs
    auauacuauaccugaccauggucuuucaauucggguuucgcgguauucgaacacguaccaauagcaguuguucguugucgucgaaaccugcuuugaaagauaguuuug         exp
    auauacuauaccugaccauggucuuucaauucggguuucgcgguauucgaacacguaccaauagcaguuguucguugucgucgaaaccugcuuugaaagauaguuuug         known
    ....................(((((((((..((((((((((((((..(((((((............).)))))).))))).)))))))))..))))))))).......   reads   mm   sample
    ..............................ucggguuucgcgguauucga..........................................................   6       0    seq
    ..............................ucggguuucgcgguauucgaaG........................................................   1       1    seq
    ..............................ucggguuucgcgguauucgaac........................................................   76      0    seq
    ..............................ucggguuucgcgguauucAaac........................................................   2       1    seq
    ..............................uNggguuucgcgguauucgaac........................................................   1       1    seq
    ..............................ucggguuucgcgguauucgaaca.......................................................   22      0    seq
    ...............................cggguuucgcgguauucgaac........................................................   1       0    seq
    ...............................cggguuucgcgguauucAaac........................................................   1       1    seq
    ...............................cggguuucgcgguauucAaaca.......................................................   1       1    seq
    ...............................cggguuucgcgguauucgaaca.......................................................   39      0    seq
    ...............................cggguuuUgcgguauucgaaca.......................................................   1       1    seq
    .....................................................................Cuucguugucgucgaaaccugcu................   1       1    seq
    ......................................................................uucguugucgucgaaacc....................   25      0    seq
    ......................................................................uucguugucgucgaaaccG...................   3       1    seq
    ......................................................................uucguuAucgucgaaaccu...................   1       1    seq
    ......................................................................uuUguugucgucgaaaccu...................   2       1    seq
    ......................................................................uucguugucgucgaaaccu...................   230     0    seq
    ......................................................................uucguugucgucAaaaccu...................   1       1    seq
    ......................................................................uucguugucguUgaaaccu...................   1       1    seq
    ......................................................................Aucguugucgucgaaaccug..................   1       1    seq
    ......................................................................uucguugucgucgaaaccug..................   50      0    seq
    ......................................................................uucguugucgucAaaaccugc.................   1       1    seq
    ......................................................................uucgAugucgucgaaaccugc.................   1       1    seq
    ......................................................................uucguugucgucgaaaccugc.................   313     0    seq
    ......................................................................uucguugucgucgaaaccAgc.................   1       1    seq
    ......................................................................Aucguugucgucgaaaccugc.................   1       1    seq
    ......................................................................uucguugucgAcgaaaccugc.................   1       1    seq
    ......................................................................uucguugucgAcgaaaccugcu................   8       1    seq
    ......................................................................uucguuGgucgaaaccugcu..................   2       1    seq
    ......................................................................uucguugucAucgaaaccugcu................   6       1    seq
    ......................................................................uucguugucgucgaaacAugcu................   7       1    seq
    ......................................................................uucguuguUgucgaaaccugcu................   18      1    seq
```

**Star** **Mature**

auauacuauaccugaccauggucuuucaauucggguuucgcgguauucgaacacguaccaauagcaguuguucguugucgucgaaaccugcuuugaaagauaguuuug

| | | | |
|---|---|---|---|
| ......................................................................uuGguugucgucgaaaccugcu................ | 2 | 1 | seq |
| ......................................................................uucguugucgucgaaaccuCcu................ | 1 | 1 | seq |
| ......................................................................uucguugucgucgaaCccugcu................ | 1 | 1 | seq |
| ......................................................................uucguugucgucgaaaccugGu................ | 4 | 1 | seq |
| ......................................................................uucguugucgucgaaacGugcu................ | 2 | 1 | seq |
| ......................................................................uucguCgucgucgaaaccugcu................ | 4 | 1 | seq |
| ......................................................................uCcguugucgucgaaaccugcu................ | 4 | 1 | seq |
| ......................................................................uucguugCcgucgaaaccugcu................ | 2 | 1 | seq |
| ......................................................................uucguugucguGgaaaccugcu................ | 2 | 1 | seq |
| ......................................................................uucguugucgucgaaaccugcG................ | 7 | 1 | seq |
| ......................................................................uucguuAucgucgaaaccugcu................ | 4 | 1 | seq |
| ......................................................................uAcguugucgucgaaaccugcu................ | 8 | 1 | seq |
| ......................................................................uucguugucgucgaaaccAgcu................ | 4 | 1 | seq |
| ......................................................................uucguugucgucgaaaccugAu................ | 4 | 1 | seq |
| ......................................................................uucguugucgucgaaaccugUu................ | 5 | 1 | seq |
| ......................................................................uucguGgucgucgaaaccugcu................ | 3 | 1 | seq |
| ......................................................................uuAguugucgucgaaaccugcu................ | 7 | 1 | seq |
| ......................................................................uucgCugucgucgaaaccugcu................ | 3 | 1 | seq |
| ......................................................................Aucguugucgucgaaaccugcu................ | 58 | 1 | seq |
| ......................................................................uucguugucgucgaaaccCgcu................ | 1 | 1 | seq |
| ......................................................................uucguuUucgucgaaaccugcu................ | 1 | 1 | seq |
| ......................................................................uucguuguAgucgaaaccugcu................ | 4 | 1 | seq |
| ......................................................................uucUuugucgucgaaaccugcu................ | 2 | 1 | seq |
| ......................................................................uucguugucgucgaaaAcugcu................ | 1 | 1 | seq |
| ......................................................................uucguugucgucgaaacUugcu................ | 6 | 1 | seq |
| ......................................................................Nucguugucgucgaaaccugcu................ | 7 | 1 | seq |
| ......................................................................uucguugucgucgaaaccuUcu................ | 2 | 1 | seq |
| ......................................................................Cucguugucgucgaaaccugcu................ | 10 | 1 | seq |
| ......................................................................uucguugucgucgaaaccuAcu................ | 3 | 1 | seq |
| ......................................................................uucguugucgucgaaUccugcu................ | 4 | 1 | seq |
| ......................................................................uucguugucgucAaaaccugcu................ | 234 | 1 | seq |
| ......................................................................uucguugucgucgaaaGcugcu................ | 2 | 1 | seq |
| ......................................................................uucguugucgucgUaaccugcu................ | 3 | 1 | seq |
| ......................................................................uucgAugucgucgaaaccugcu................ | 12 | 1 | seq |
| ......................................................................uucgGugucgucgaaaccugcu................ | 2 | 1 | seq |
| ......................................................................uucguugucguUgaaaccugcu................ | 18 | 1 | seq |
| ......................................................................Gucguugucgucgaaaccugcu................ | 12 | 1 | seq |
| ......................................................................uucguugGcgucgaaaccugcu................ | 1 | 1 | seq |
| ......................................................................uucguugucgucCaaaccugcu................ | 3 | 1 | seq |
| ......................................................................uucguugucgucgGaaccugcu................ | 4 | 1 | seq |
| ......................................................................uucguugucgucgaaGccugcu................ | 3 | 1 | seq |
| ......................................................................uucguAgucgucgaaaccugcu................ | 5 | 1 | seq |
| ......................................................................uucguugucgucgaGaccugcu................ | 3 | 1 | seq |
| ......................................................................uucguugucgucgaaaUcugcu................ | 1 | 1 | seq |
| ......................................................................uucguugucgucgaaaccugcC................ | 22 | 1 | seq |
| ......................................................................uucguugucgucgaaaccugcA................ | 31 | 1 | seq |
| ......................................................................uucguugAcgucgaaaccugcu................ | 12 | 1 | seq |
| ......................................................................uucguugucguAgaaaccugcu................ | 6 | 1 | seq |
| ......................................................................uucguugucgCcgaaaccugcu................ | 2 | 1 | seq |
| ......................................................................uucguugucgucgaaaccugcu................ | 22230 | 0 | seq |
| ......................................................................uuUguugucgucgaaaccugcu................ | 62 | 1 | seq |
| ......................................................................uucguugucgucgaUaccugcu................ | 1 | 1 | seq |
| ......................................................................uucAuugucgucgaaaccugcu................ | 5 | 1 | seq |
| ......................................................................uucgAugucgucgaaaccugcuu............... | 3 | 1 | seq |
| ......................................................................uucguugucgucgaaaccugcuC............... | 1 | 1 | seq |
| ......................................................................uucguugucgucgaaacUugcuu............... | 1 | 1 | seq |
| ......................................................................uucgCugucgucgaaaccugcuu............... | 2 | 1 | seq |
| ......................................................................uucguugucgucgaaaccugcuu............... | 4185 | 0 | seq |
| ......................................................................uucguugucgucgaaaAcugcuu............... | 2 | 1 | seq |
| ......................................................................uucguugAcgucgaaaccugcuu............... | 2 | 1 | seq |
| ......................................................................uucguugucgucgaaUccugcuu............... | 1 | 1 | seq |
| ......................................................................uucguugucgucgaaaccugcCu............... | 972 | 1 | seq |
| ......................................................................uucguugucgucgaaaccugGuu............... | 1 | 1 | seq |
| ......................................................................Gucguugucgucgaaaccugcuu............... | 4 | 1 | seq |
| ......................................................................uucguugucgucgaGaccugcuu............... | 1 | 1 | seq |
| ......................................................................uucguugCcgucgaaaccugcuu............... | 1 | 1 | seq |
| ......................................................................uucguugucgucgaaaccugcuA............... | 50 | 1 | seq |
| ......................................................................uucguCgucgucgaaaccugcuu............... | 1 | 1 | seq |
| ......................................................................uucAuugucgucgaaaccugcuu............... | 1 | 1 | seq |
| ......................................................................uucguugucgucgaaacAugcuu............... | 1 | 1 | seq |

```
                              Star                                    Mature
auauacuauaccugaccauggucuuucaauucggguuucgcgguauucgaacacguaccaauagcaguuguucguugucgucgaaaccugcuuugaaagauaguuuug
......................................................................uucguugucgucgaaaccugUuu...............   2     1     seq
......................................................................uucguugucguUgaaaccugcuu...............   4     1     seq
......................................................................uucguugucguAgaaaccugcuu...............   1     1     seq
......................................................................Nucguugucgucgaaaccugcuu...............   2     1     seq
......................................................................uuAguugucgucgaaaccugcuu...............   2     1     seq
......................................................................uuUguugucgucgaaaccugcuu...............   5     1     seq
......................................................................uucguugucgucgaaaccAgcuu...............   1     1     seq
......................................................................uCcguugucgucgaaaccugcuu...............   1     1     seq
......................................................................uucCuugucgucgaaaccugcuu...............   1     1     seq
......................................................................uucguAgucgucgaaaccugcuu...............   2     1     seq
......................................................................uucguugucgucAaaaccugcuu...............   32    1     seq
......................................................................uucguugucgCcgaaaccugcuu...............   1     1     seq
......................................................................Aucguugucgucgaaaccugcuu...............   13    1     seq
......................................................................uucguugucgucgaaaccugcuuu..............   167   0     seq
......................................................................uucguugucgucgaaaccugcCuu..............   1     1     seq
......................................................................uucguugucgucgaaaccugcuuA..............   17    1     seq
......................................................................uucguugucgucgaaaccugcuAu..............   12    1     seq
......................................................................uucguugucgucgaaaccugcuuC..............   1     1     seq
......................................................................uucguugucgucAaaaccugcuuu..............   1     1     seq
......................................................................Aucguugucgucgaaaccugcuuu..............   1     1     seq
......................................................................uucguugucgucgaaaccugcuuuA.............   3     1     seq
......................................................................uucguugucgucgaaaccugcuuuU.............   13    1     seq
......................................................................uucguugucgucgaaaccugcuuug.............   2     0     seq
.......................................................................uUguugucgucgaaaccugcu................   1     1     seq
.........................................................................guugucgucgaaaccugcu................   2     0     seq
```
